# T-Cell Tropism of Simian Varicella Virus during Primary Infection

**DOI:** 10.1371/journal.ppat.1003368

**Published:** 2013-05-09

**Authors:** Werner J. D. Ouwendijk, Ravi Mahalingam, Rik L. de Swart, Bart L. Haagmans, Geert van Amerongen, Sarah Getu, Don Gilden, Albert D. M. E. Osterhaus, Georges M. G. M. Verjans

**Affiliations:** 1 Department of Viroscience, Erasmus MC, Rotterdam, the Netherlands; 2 Department of Neurology, University of Colorado School of Medicine, Aurora, Colorado, United States of America; 3 Department of Microbiology, University of Colorado School of Medicine, Aurora, Colorado, United States of America; University of Pittsburgh, United States of America

## Abstract

Varicella-zoster virus (VZV) causes varicella, establishes a life-long latent infection of ganglia and reactivates to cause herpes zoster. The cell types that transport VZV from the respiratory tract to skin and ganglia during primary infection are unknown. Clinical, pathological, virological and immunological features of simian varicella virus (SVV) infection of non-human primates parallel those of primary VZV infection in humans. To identify the host cell types involved in virus dissemination and pathology, we infected African green monkeys intratracheally with recombinant SVV expressing enhanced green fluorescent protein (SVV-EGFP) and with wild-type SVV (SVV-wt) as a control. The SVV-infected cell types and virus kinetics were determined by flow cytometry and immunohistochemistry, and virus culture and SVV-specific real-time PCR, respectively. All monkeys developed fever and skin rash. Except for pneumonitis, pathology produced by SVV-EGFP was less compared to SVV-wt. In lungs, SVV infected alveolar myeloid cells and T-cells. During viremia the virus preferentially infected memory T-cells, initially central memory T-cells and subsequently effector memory T-cells. In early non-vesicular stages of varicella, SVV was seen mainly in perivascular skin infiltrates composed of macrophages, dendritic cells, dendrocytes and memory T-cells, implicating hematogenous spread. In ganglia, SVV was found primarily in neurons and occasionally in memory T-cells adjacent to neurons. In conclusion, the data suggest the role of memory T-cells in disseminating SVV to its target organs during primary infection of its natural and immunocompetent host.

## Author Summary

Varicella-zoster virus (VZV) causes varicella, establishes life-long latent infection in ganglia and reactivates later in life to cause zoster. VZV is acquired via the respiratory route, with skin rash occurring up to 3 weeks after exposure. The cell types that transport VZV to skin and ganglia during primary infection are unknown. Simian varicella virus (SVV) infection of non-human primates mimics clinical, pathological and immunological features of human VZV infection. African green monkeys were infected with recombinant SVV expressing enhanced green fluorescent protein (SVV-EGFP) or wild-type SVV (SVV-wt) as a control. By visualizing SVV-EGFP−infected cells in the living animal and in tissue samples, we identified the virus-infected cell types in blood, lungs, skin and ganglia during primary infection. Our data demonstrate that during viremia, SVV predominantly infects peripheral blood memory T-cells. Detection of SVV-infected memory T-cells in lungs, in early varicella skin lesions and also, albeit to a lesser extent, in ganglia suggests a role for memory T-cells in transporting virus to these organs. Our study provides novel insights into the cell types involved in virus dissemination and the overall pathology of varicella in a non-human primate model.

## Introduction

Varicella-zoster virus (VZV) is a ubiquitous human neurotropic alphaherpesvirus that causes varicella (chickenpox) as a primary infection and herpes zoster (shingles) upon reactivation of latent virus [1]. Primary VZV infection is acquired via the respiratory route and varicella occurs 2–3 weeks after exposure [2], [3]. The pathogenesis of varicella is largely unknown, mostly due to the prolonged incubation period and restricted host range of the virus. VZV is detected in lymphocytes of varicella patients [4], suggesting that the virus spreads to susceptible organs including skin and ganglia via a cell-associated viremia [4]. However, the low number of VZV-infected lymphocytes has precluded their identification during natural infection in humans [5].

Most of the current understanding of VZV pathogenesis is based on experimental infection of human fetal tissue transplanted in severe combined immunodeficient mice (SCID-hu model) [6], [7]. In this model, VZV has a tropism for T-cells within thymus and liver xenografts [8]. It has been postulated that VZV initially replicates in respiratory epithelial cells and is transferred to T-cells within tonsilar lymphoid tissue contacting the upper respiratory tract [9], [10]. Virus transport to human fetal skin and ganglia explants in SCID-hu mice can be mediated by T-cells [11], [12], most likely activated memory CD4 T-cells expressing the skin homing markers C-C type chemokine receptor type 4 (CCR4) and cutaneous lymphocyte antigen (CLA) [10]. However, the VZV SCID-hu mouse model does not reproduce the complex and dynamic virus-host interactions involved in the dissemination of VZV to its target organs during primary infection in its natural and immunocompetent host [6], [7].

Simian varicella virus (SVV) produces a naturally occurring disease in non-human primates with clinical, pathological and immunological features that parallel human VZV infection [13], [14]. The prevalence of SVV in free-ranging non-human primates is largely unknown. However, SVV outbreaks in primate centers have been associated with the introduction of monkeys captured from the wild into the colony [15]. The genomes of SVV and VZV are similar in size, structure and genetic organization, with an estimated 70–75% DNA homology [16]. SVV causes varicella, becomes latent in ganglionic neurons and reactivates after stress and immunosuppression to cause herpes zoster [17], [18]. A cell-associated viremia is detected from 3 days post-infection (dpi), with the highest number of infected lymphocytes just before the onset of skin rash [14], [19]. SVV reaches the ganglia before skin rash [20], [21], indicating viremic spread to ganglia.

The aim of the present study was to characterize the kinetics of virus infection and the cell types involved in the dissemination of SVV during primary infection. We have previously shown that infection of macaques with recombinant measles virus expressing EGFP (rMV-EGFP) facilitated the identification of the cell types involved in MV pathogenesis with unprecedented sensitivity [22], [23], [24], [25]. To detect SVV-infected cells at the low frequencies expected in blood and lungs, we infected African green monkeys (AGMs) with recombinant SVV expressing EGFP (SVV-EGFP) and, as a control, wild-type SVV (SVV-wt) to study SVV pathogenesis at the whole organism, tissue and cellular levels in its natural and immunocompetent host. The data presented suggest a crucial role for memory T-cells in the dissemination of SVV during primary infection.

## Results/Discussion

### SVV-infected African green monkeys develop transient fever and skin rash

**Figure 1:**
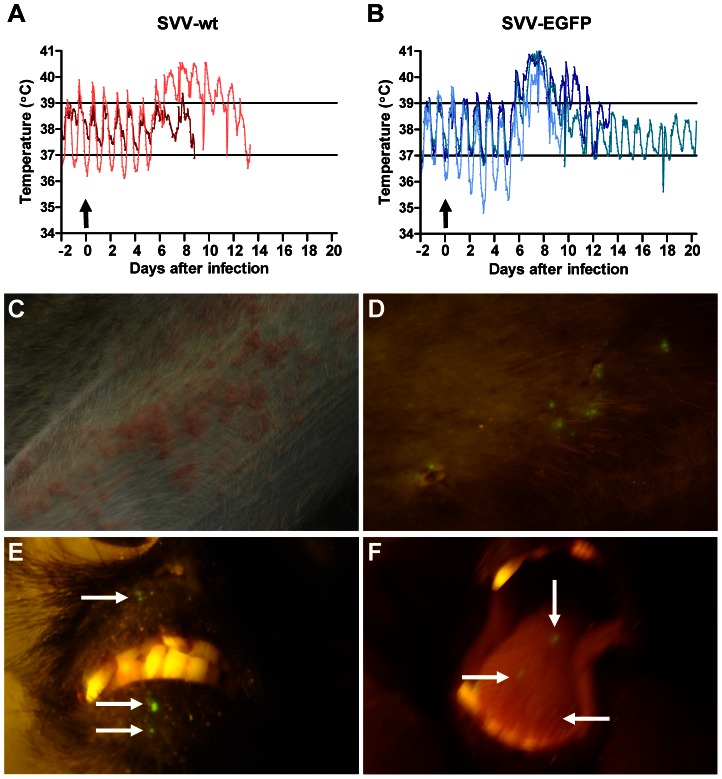
Experimental SVV infection of African green monkeys results in transient fever and skin rash. (A, B) Fluctuations in body temperature after infection with SVV-wt and SVV-EGFP, respectively, were measured by intraperitoneally implanted temperature transponders during primary infection. Arrows indicate time of SVV inoculation; horizontal lines indicate normal range in body temperature before infection. (C) Vesicular skin rash at 8 dpi with SVV-wt. (D) Macroscopic detection of EGFP fluorescence on skin at 8 dpi with SVV-EGFP. (E) Macroscopic detection of EGFP fluorescence (arrows) on lips at 9 dpi with SVV-EGFP. (F) Macroscopic detection of EGFP-positive lesions (arrows) on tongue at 8 dpi with SVV-EGFP.

Five SVV-seronegative adult AGMs were infected intratracheally with SVV-wt (n = 2) or SVV-EGFP (n = 3). A transient increase in body temperature was seen between 6 and 11 dpi (Fig. 1A and B). SVV-wt−infected animals developed skin rash starting at 6 dpi, which increased in severity until 9 to 10 dpi and resolved thereafter (Fig. 1C). Macroscopic EGFP fluorescent lesions were detected on the skin and lips of all SVV-EGFP−infected animals starting at 7 dpi, which increased in severity until 9 dpi and resolved by 13 dpi (Fig. 1D and E). EGFP fluorescent lesions were also detected on the tongue of SVV-EGFP−infected monkeys, coinciding with appearance of skin rash (Fig. 1F). No lesions were observed on the lips and tongues of SVV-wt−infected animals, demonstrating the increased sensitivity of using SVV-EGFP to study varicella pathogenesis. Skin rash was more severe in SVV-wt− compared to SVV-EGFP−infected monkeys. Collectively, the findings indicate the close resemblance of the clinical signs associated with experimental SVV-EGFP infection of AGMs and those of primary VZV infection in humans.

### SVV infection of alveolar myeloid cells and T-cells in the lung

**Figure 2:**
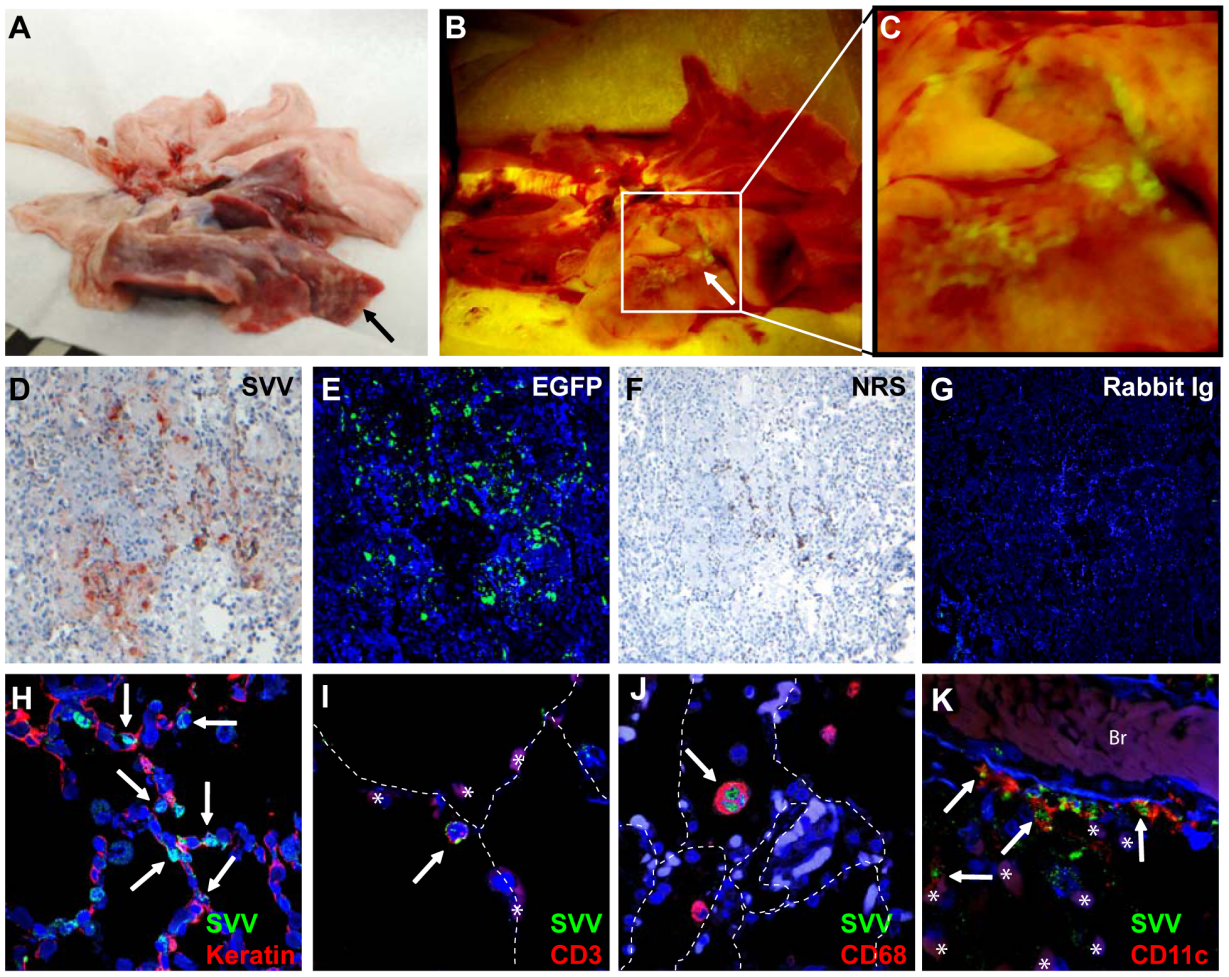
Macroscopic and microscopic detection of SVV-infected cells in lungs of infected African green monkeys. (A) Macroscopic appearance of consolidated dark-red lesions (black arrow) in the lung of an SVV-wt−infected monkey at 13 dpi. (B) Macroscopic detection of EGFP fluorescence in affected area of lung (white arrow) of an SVV-EGFP-infected monkey at 9 dpi. (C) Magnification of the affected area in panel B shows EGFP fluorescence. (D–G) Serial lung sections obtained from an SVV-EGFP−infected monkey at 9 dpi analyzed by immunohistochemistry (IHC) for SVV antigens (D) or by immunofluorescence (IF) for EGFP (E), with two sections analyzed by IHC (F) or IF (G) using normal rabbit serum (NRS) and isotype control antibodies, respectively. Lung sections obtained from an SVV-wt−infected monkey at 9 dpi were analyzed using dual IF for SVV (green) and: cytokeratin (red) (H), CD3 (red) (I), CD68 (red) (J), and CD11c (red) (K) antigens. Arrows indicate double-positive cells. Asterisks indicate autofluorescent erythrocytes. Dashed lines indicate alveolar septa. Br: bronchus. Nuclei were counterstained with DAPI. D–G: 100× magnification; H, J: 400× magnification; I, K: 400× magnification and 2× digital zoom.

All SVV-infected animals became dyspneic at the time of skin rash. Macroscopic examination of lungs showed multifocal pulmonary consolidation and hemorrhage affecting at least one lobe in all animals euthanized 9 or 13 dpi (Fig. 2A). Diffuse EGFP fluorescence was detected in an SVV-EGFP−infected monkey at 9 dpi (Fig. 2B and C). Combined immunohistochemical (IHC) and immunofluorescence (IF) analyses for SVV antigens and EGFP on consecutive sections of lung showed that EGFP expression was restricted to SVV antigen-positive cells (Fig. 2D–G), demonstrating that EGFP is a valid marker to identify SVV-infected cells in the monkeys. To investigate SVV-infected cell types *in situ*, lung tissue sections were analyzed by dual-IF staining with SVV-specific antiserum and anti-keratin, -CD3, -CD68 and -CD11c mouse monoclonal antibodies (mAbs). SVV-infected cells were readily detected in lungs at 9 dpi, but not at later times (data not shown). At 9 dpi, abundant SVV^pos^keratin^pos^ lung epithelial cells were observed (Fig. 2H), as well as SVV^pos^CD3^pos^ T-cells (Fig. 2I). In addition, SVV antigens were found in intra-alveolar cells that co-expressed CD68 and/or CD11c, consistent with alveolar macrophages (AM), some of which appeared to have phagocytosed SVV-infected cells (Fig. 2J). Occasionally, SVV^pos^CD11c^pos^ dendritic cell (DC)-like cells displaying multiple branched projections were observed adjacent to bronchi (Fig. 2K).

**Figure 3:**
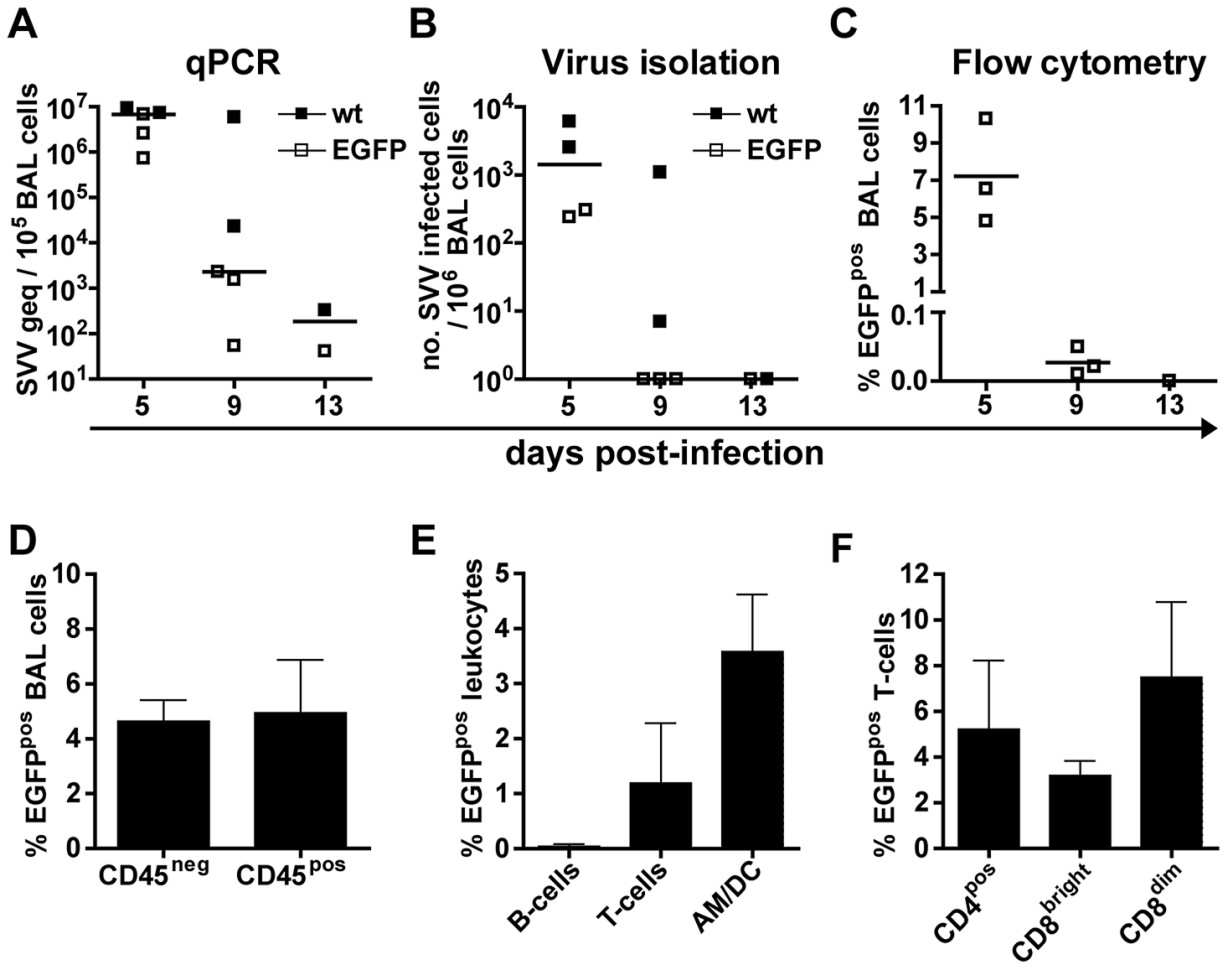
SVV preferentially infects myeloid cells and T-cells in lungs of infected African green monkeys. (A) Bronchoalveolar lavage (BAL) cells obtained at 5, 9 and 13 dpi were analyzed for viral DNA by SVV-specific real-time qPCR. Data are expressed as genome equivalent copies (geq) per 10^5^ BAL cells. (B) BAL cells were analyzed for infectious virus by co-cultivation with BSC-1 cells. (C–F) Percentage of EGFP-positive cells as assessed by flow cytometry in: all BAL cells (C); leukocytes (CD45^pos^ cells), non-leukocytes (CD45^neg^ cells) from BAL samples (D) and leukocyte subsets within BAL (E). Leukocyte subsets were identified based on the differential expression of the following markers: AM/DC = CD45^pos^CD3^neg^CD20^neg^MHC-II^pos^CD14^pos/dim^, T-cells = CD45^pos^CD3^pos^, B-cells = CD45^pos^CD20^pos^MHC-II^pos^; and the indicated T-cell subsets (F). AM/DC are BAL-derived lymphocytes expressing markers shared by dendritic cells (DC) and alveolar macrophages (AM). Horizontal bars indicate median values. (D–F) BAL cells were obtained at 5 dpi and data are given as means ± SEM.

To define the kinetics of virus replication and the cell types infected in the respiratory tract during primary SVV infection, bronchoalveolar lavage (BAL) cells were obtained at 5 dpi, 9 dpi and at necropsy (9, 13 or 20 dpi). SVV DNA load and infectious virus titers in BAL cells peaked at 5 dpi and declined rapidly thereafter (Fig. 3A and B). Infectious virus was not recovered from BAL cells at 13 and 20 dpi (Fig. 3B and data not shown). The viral DNA load and infectious SVV titer in BAL samples were similar in SVV-wt− and SVV-EGFP−infected monkeys at 5 dpi, indicating a similar level of replication of both viruses in lung.

At 5 dpi, 7.2% of BAL cells from SVV-EGFP−infected monkeys were EGFP^pos^ (Fig. 3C). In EGFP^pos^ BAL cells, equal numbers of CD45^pos^ (leukocytes) and CD45^neg^ cells, most likely bronchial and alveolar epithelial cells, were detected (Fig. 3D and Fig. S1). CD45^pos^ BAL cells could be categorized as T-cells, B-cells and alveolar myeloid cells, i.e. large granular cells expressing high levels of both CD14 and HLA-DR (Fig. S1). These myeloid cells could be alveolar macrophages (AM) and/or DC. At 5 dpi, 82% of CD45^pos^ BAL cells were alveolar myeloid cells, 17% were T-cells and only 1% were B-cells (data not shown). Most EGFP^pos^CD45^pos^ BAL cells were alveolar myeloid cells and T-cells (Fig. 3E). CD4^pos^, CD8^dim^ and CD8^bright^ T-cells were infected at equal frequencies (Fig. 3F). The number of BAL-derived T-cells was too low to determine their differentiation status unequivocally (data not shown). At 9 dpi, frequencies of EGFP^pos^CD45^pos^ BAL cells were too low to conclusively identify the SVV-infected leukocyte subsets (Fig. 3C).

### SVV infection of memory T-cells in blood during viremia

**Figure 4:**
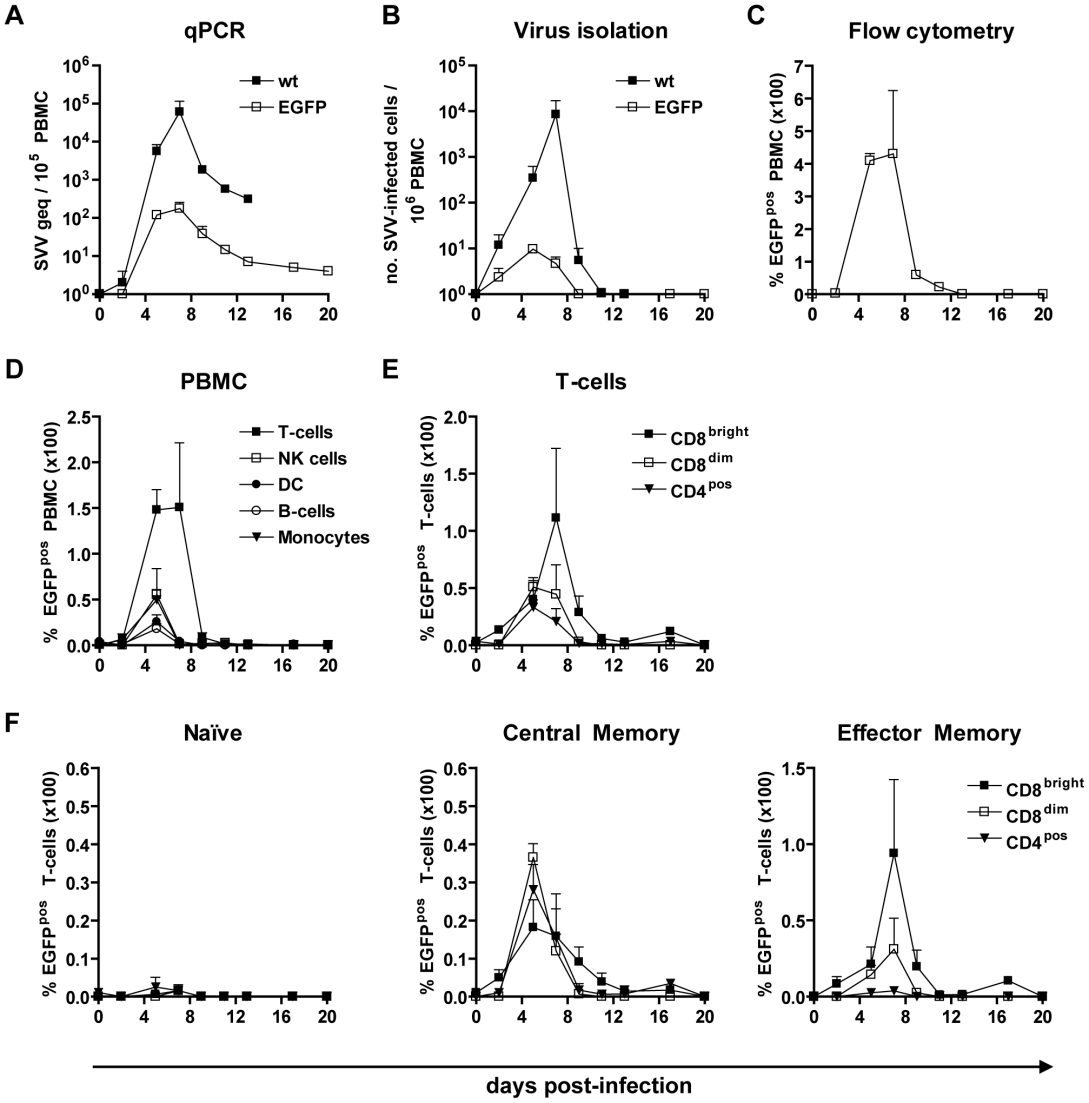
SVV infects predominantly memory T-cells in blood after infection in African green monkeys. (A) Average SVV DNA load in PBMC of SVV-wt− (closed squares) and SVV-EGFP− (open squares) infected monkeys determined by SVV-specific real-time qPCR. (B) PBMC from SVV-wt− (closed squares) and SVV-EGFP− (open squares) infected monkeys were analyzed for infectious SVV by co-cultivation with BSC-1 cells. (C) PBMC from SVV-EGFP−infected monkeys were analyzed for EGFP expression by flow cytometry. (D) EGFP expression in PBMC subsets from SVV-EGFP−infected monkeys. Data are given as percentage of EGFP^pos^ cells within each lymphocyte subset relative to the total number of PBMC, as determined by flow cytometry. Lymphocyte subsets were defined by differential expression of the following markers: T-cells = CD3^pos^CD16^neg^ cells, B-cells = CD20^pos^MHC-II^pos^cells, natural killer (NK) cells = CD3^neg^CD16^pos^ cells, dendritic cells (DC) = CD3^neg^CD14^neg^CD16^neg^CD20^neg^CD14^neg^MHC-II^pos^ cells, and monocytes = CD3^neg^CD14^pos^MHC-II^pos^ cells. (E and F) Percentage of EGFP^pos^ cells among each T-cell subset relative to the number of CD8^bright^, CD8^dim^ and CD4^pos^ T-cells (E) and in naive, central memory and effector memory T-cells (F) from SVV-EGFP−infected monkeys as evaluated by flow cytometry. In all panels, data are means ± SEM.

To determine the kinetics of virus infection and identify the blood lymphocyte subsets infected during the viremic phase of varicella, peripheral blood mononuclear cells (PBMC) isolated at multiple dpi from SVV-infected monkeys were analyzed. SVV DNA was detected in PBMC from 2 dpi until necropsy (Fig. 4A). Viral DNA load in PBMC peaked at 7 dpi and was higher in SVV-wt− compared to SVV-EGFP−infected monkeys (Fig. 4A). Infectious virus was isolated from PBMC of both SVV-EGFP− and SVV-wt−infected monkeys until 9 and 11 dpi (Fig. 4B). EGFP^pos^ lymphocytes were detected from 5 to 11 dpi, peaking at 7 dpi (Fig. 4C). Together, the data indicate that the kinetics of viral DNA load and infectious virus titer represent the temporal change in the number of circulating SVV-infected lymphocytes, but not in the replication of SVV in blood lymphocytes during viremia. The rapid loss of SVV-infected lymphocytes from the circulation could be caused by virus-induced apoptosis [26] or, alternatively, infected lymphocytes may be cleared from the circulation by the SVV-specific adaptive immune response [14], [21], [27].

At 5 dpi, EGFP^pos^ cells were detected at similar frequencies in all major PBMC subsets (i.e., T-cells, B-cells, natural killer cells, monocytes and dendritic cells) (Fig. 4D and Fig. S2). However, given that most PBMC are T-cells (Fig. S2), T-cells were identified as the main SVV-infected lymphocyte subset in blood (Fig. 4D). Moreover, at the peak of viremia (7 dpi) T-cells were the only SVV-infected cells demonstrated in blood. Unlike humans and macaques, AGMs have three distinct T-cell subsets: CD8^bright^, CD8^dim^ and CD4^pos^ T-cells (Fig. S2) [28]. While CD8^bright^ T-cells correspond to classical human CD8+ T-cells, CD4^pos^ T-cells and CD8^dim^ T-cells are considered dynamic populations of AGM T-helper cells functionally equivalent to human CD4+ T-cells [28]. Similar levels of CD8^bright^, CD8^dim^ and CD4^pos^ T-cells were SVV-infected, most of which were memory T-cells (Fig. 4E and F). Importantly, at 5 and 7 dpi, predominantly central memory (CM) T-cells and effector memory (EM) T-cells, respectively, were infected (Fig. 4F). The apparent dual phase of SVV-infected CM and EM T-cells may reflect the organ in which the T-cells have been infected. CM T-cells are preferentially found in lymphoid tissues, whereas EM T-cells are migratory memory T-cells that home to peripheral tissues to orchestrate local immune responses and may ultimately function as tissue-resident T-cells to sense the cognate antigen locally for extended periods of time [29], [30]. CM T-cells may have been infected in lymphoid tissues and EM T-cells in lungs. Alternatively, SVV infection might have altered the expression of membrane markers used herein to identify AGM-derived CM and EM T-cells. Finally, virus infection may have induced differentiation of CM T-cells into EM T-cells *in vivo*.

*In vitro* infection studies on human tonsil-derived lymphocytes showed that VZV preferentially infects T-cells expressing the activation marker CD69 and skin-homing markers CCR4 and CLA [10]. To address this issue in SVV-EGFP−infected monkeys, peripheral blood-derived EGFP^pos^ T-cells obtained at 5 and 7 dpi were analyzed for expression of both CCR4 and the T-cell activation marker CD137, the latter marker is selectively expressed by T-cells early after recognition of their cognate antigen [31], [32]. No preference of SVV for memory T-cells expressing CCR4 or CD137 was seen *in vivo* (Fig. S3), suggesting that SVV did not infect virus-specific T-cells that recognized SVV-infected antigen presenting cells like macrophages or DCs.

To determine whether the predominant infection of memory T-cells *in vivo* reflects viral tropism for a specific lymphocyte subset, PBMC from SVV-naive AGMs were infected *in vitro* with SVV-EGFP. Expression of EGFP was restricted to lymphocytes that expressed SVV antigens (Fig. S4A), supporting the use of EGFP as a surrogate marker for SVV-infected cells in flow cytometry. While all major PBMC subsets appeared to be equally susceptible to SVV infection, T-cells were the prominent SVV-infected PBMC subset *in vitro* (Fig. S4B), with similar infection levels in CD4^pos^, CD8^dim^ and CD8^bright^ T-cells (Fig. S4C). In particular, significantly more memory T-cells were infected compared to naive T-cells (*p*<0.05; Mann-Whitney test) (Fig. S4D). Thus, SVV preferentially infects memory T-cells rather than naive T-cells both *in vivo* (Fig. 4) and *in vitro* (Fig. S4).

### Detection of SVV in lymphoid organs

**Figure 5:**
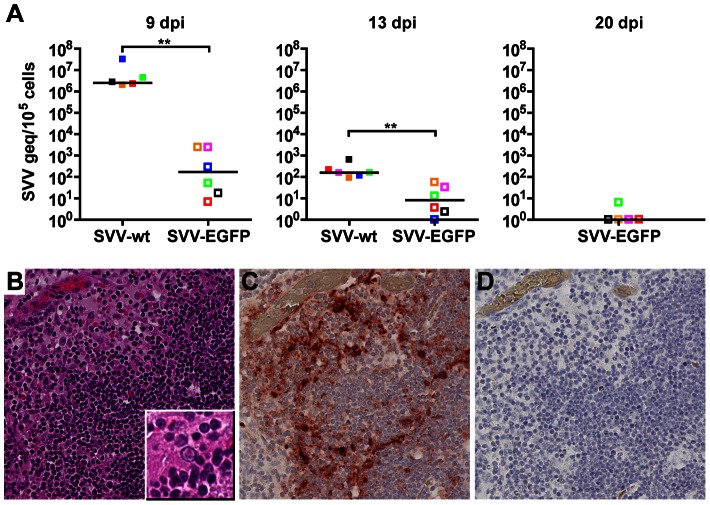
Detection of SVV in lymphoid organs from infected African green monkeys. (A) Real-time qPCR analysis of SVV DNA load in tonsil, lymph nodes and spleen from SVV-wt− (closed squares) and SVV-EGFP− (open squares) infected monkeys at 9, 13 and 20 dpi. Squares indicate individual tissues, i.e., tonsils (red), tracheobronchial lymph nodes (LN) (green), axillary LN (pink), mandibular LN (blue), inguinal LN (orange) and spleen (black). Horizontal bar indicates the median value. (B–D) Serial sections of tonsil from an SVV-wt−infected monkey stained with hematoxylin and eosin (inset shows a Cowdry type A intranuclear inclusion body) (B) or examined immunohistochemically using rabbit anti-SVV antibodies (C) or control normal rabbit serum (D). Magnification: 200×. The area of tonsils containing multiple intranuclear inclusion bodies contained numerous cells expressing SVV protein. **p<0.01 by Mann-Whitney test.

Alveolar macrophages and lung-resident DC transport antigens to lung-draining lymph nodes for presentation to T-cells [33], [34], and VZV-infected human DCs can transfer infectious virus to T-cells *in vitro* [35]. We hypothesized that SVV-infected alveolar myeloid cells transport SVV to draining lymph nodes for subsequent virus transfer to memory T-cells. High SVV DNA loads were detected in lymph nodes, tonsils and spleens of SVV-infected monkeys at 9 dpi, declining rapidly thereafter (Fig. 5A). Cells in lymph nodes and tonsils of SVV-infected monkeys contained intranuclear inclusions bodies and SVV antigen (Fig. 5B and C). Tracheobronchial lymph nodes showed more pronounced SVV-induced histopathology compared to peripheral lymph nodes (data not shown). However, SVV DNA loads were comparable in different lymph nodes collected at 9 dpi (Fig. 5A), emphasizing the need to investigate lymph nodes at earlier times after infection. In addition, detection of SVV-infected memory T-cells in blood may represent lung-resident T-cells involved in SVV dissemination. SVV infects alveolar epithelial cells leading to alveolar wall damage (data not shown) [19], [27], [36], which may result in egress of SVV-infected T-cells into the circulation.

### SVV-infected perivascular lymphocytes in early varicella lesions implicate hematogenous spread of SVV to the skin

**Figure 6:**
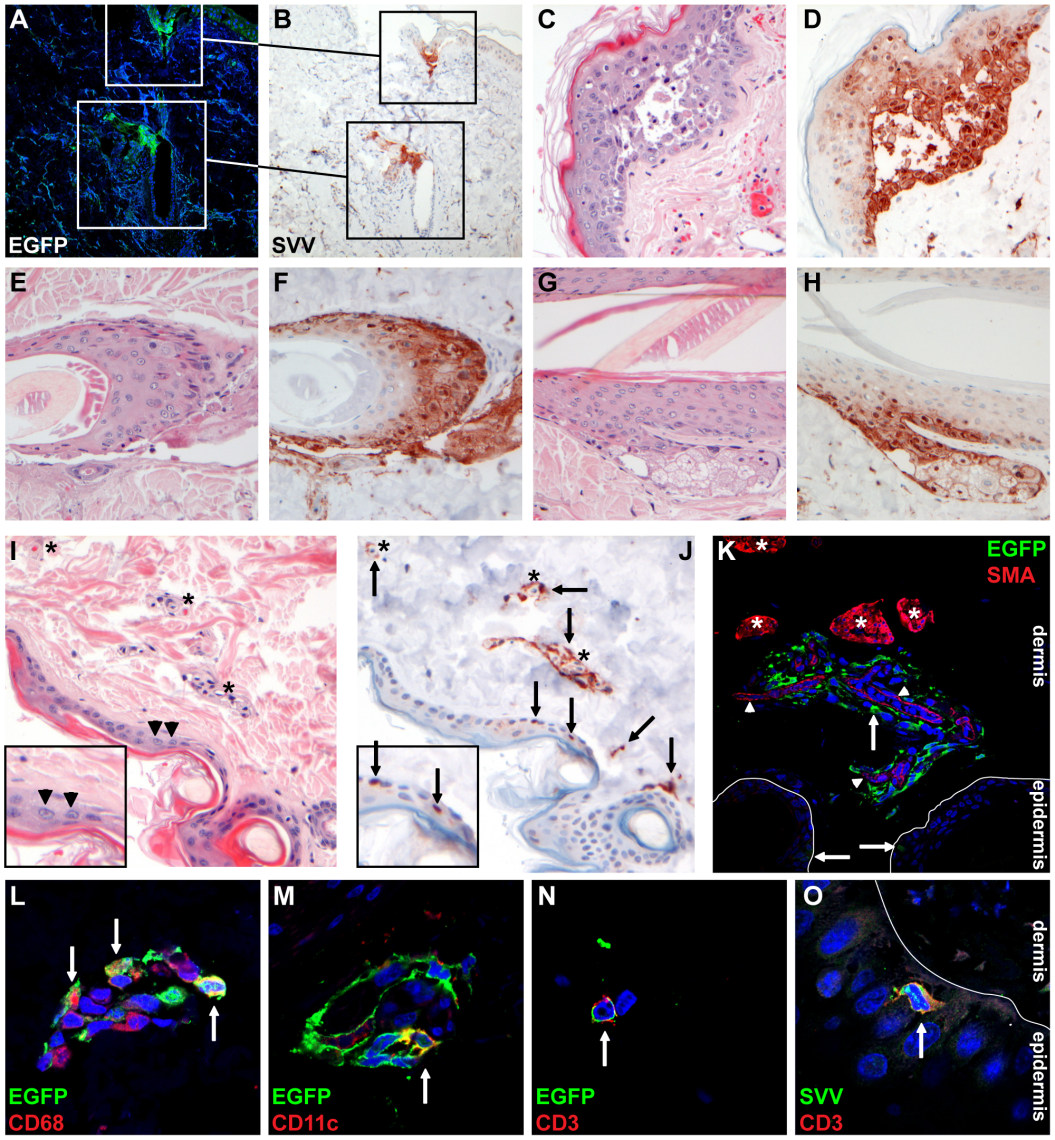
Detection of SVV-infected cells in varicella skin lesions from infected African green monkeys. (A, B) Consecutive sections of skin obtained from an SVV-EGFP-infected monkey at 9 dpi and stained by immunofluorescence (IF) for EGFP (A) and by immunohistochemistry (IHC) for SVV antigens (B) show co-localization of SVV proteins and EGFP. Squares indicate the same area of tissue. (C–H) Consecutive sections of skin obtained from an SVV-wt−infected animal at 9 dpi and examined by staining with hematoxylin and eosin (H&E) or by IHC for SVV show virus-induced histopathology and viral proteins in epidermal blisters (C and D), dermal hair follicles (E and F) and dermal sebaceous glands (G and H). (I, J) Consecutive skin sections obtained from an SVV-EGFP-infected monkey at 9 dpi and stained with H&E (I) or by IHC for SVV antigens (J) show blood vessels (asterisks) surrounded by SVV protein-positive cells (arrows). Inset: magnification of the epidermis showing Cowdry type A intranuclear inclusion bodies in panel I (arrowheads) and SVV protein-positive cells in panel J (arrows). (K) Skin section from an SVV-EGFP-infected animal obtained at 9 dpi and double-stained for EGFP (green) and alpha-smooth muscle actin (SMA; red). Asterisks indicate SMA-positive sweat glands, arrowheads indicate SMA-positive blood vessels, and arrows indicate EGFP-positive cells. (L–N) Skin sections obtained at 9 dpi and double-stained for EGFP (green) and: CD68 (red) (L); CD11c (red) (M); and CD3 (red) (N). Arrows indicate dual-stained cells. (O) Skin section obtained at 9 dpi and double-stained for SVV (green) and CD3 (red). Arrows indicate dual-stained cells. A, B: 100× magnification; C–K: 200× magnification; L–O: 400× magnification and 2× digital zoom.

Detailed *in situ* analysis was performed to identify the SVV-infected cell types in varicella skin lesions. Macroscopic detection of EGFP fluorescence corresponded to SVV infection of the skin, as demonstrated by the co-localization of SVV protein and EGFP in consecutive skin sections obtained from an SVV-EGFP–infected monkey (Fig. 6A and B). In vesicular skin lesions, SVV predominantly infected keratinocytes (Fig. 6C and D). In deeper skin layers, SVV protein was frequently detected in hair follicles (Fig. 6E and F) and sebaceous glands (Fig. 6G and H).

Analysis of skin biopsies from SVV-EGFP−infected monkeys allowed investigation of the early stages of varicella, as evidenced on the skin by the appearance of EGFP fluorescent areas in the absence of lesions visible to the naked eye. In these biopsies, SVV protein expression was consistently located within perivascular lymphocytes (Fig. 6I–K). Dual-IF staining for EGFP and specific lymphocyte markers identified SVV-infected perivascular cell subsets as CD68^pos^ macrophages (Fig. 6L), CD11c^pos^ DCs (Fig. 6M) and CD3^pos^ T-cells (Fig. 6N). The remaining SVV-infected cells, which stained negative for lymphocyte markers, phenotypically resembled dendrocytes (data not shown) [37]. Interestingly, SVV-infected T-cells were also observed in the epidermis of SVV-wt infected monkeys at 9 dpi (Fig. 6O). Flow cytometric analysis of skin-resident T-cells showed exclusively memory T-cells, mostly EM T-cells (data not shown).

Collectively, these data suggest that SVV reaches the skin hematogenously. Since the skin vasculature is composed of an upper horizontal superficial vascular plexus just beneath the epidermal surface and a deep vascular plexus that supplies the hair bulbs and sweat glands [38], it seems likely that SVV-infected memory T-cells transfer the virus to skin-resident perivascular macrophages, DCs or dendrocytes, which in turn transfer SVV to adjacent epidermal or hair follicle keratinocytes via cell-to-cell spread. Alternatively, epidermal SVV-infected T-cells may transfer the virus directly to skin epithelial cells (Fig. 6O).

### Neurons are the main SVV-infected cell types in ganglia

**Figure 7:**
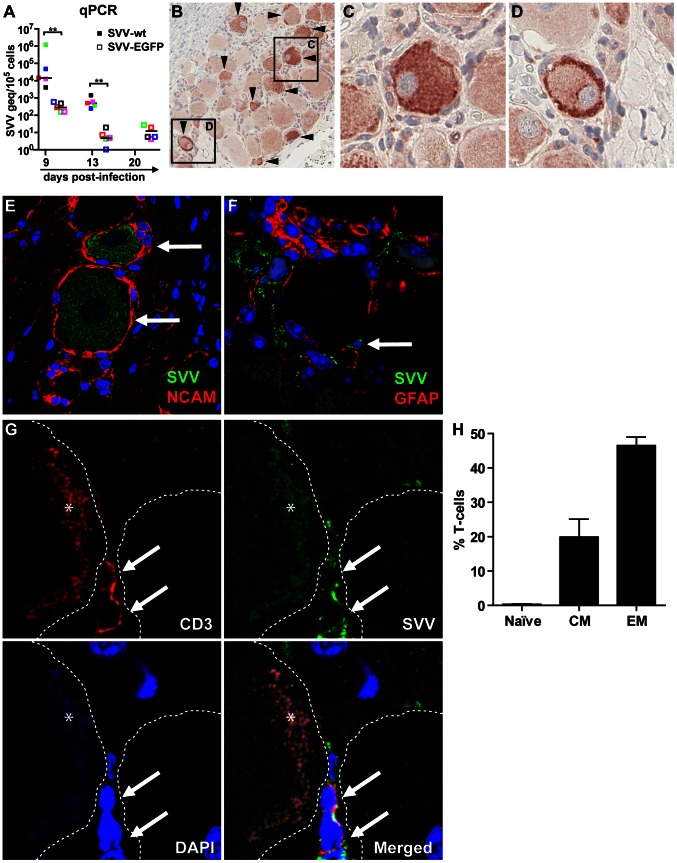
Detection of SVV-infected cells in ganglia of infected African green monkeys. (A) Virus DNA load was determined in ganglia at 9, 13 and 20 dpi by SVV-specific real-time qPCR. Filled and open squares represent pooled ganglia from the same level of the neuraxis from animals infected with SVV-wt and SVV-EGFP, respectively. Colors indicate level of the neuraxis: trigeminal (black), cervical (red), thoracic (blue), lumbar (green) and sacral (pink) ganglia. Horizontal bars represent mean viral DNA load per animal. (B) Immunohistochemical detection of SVV proteins (arrowheads) in a cervical ganglion at 9 dpi. Squares indicate corresponding tissue areas shown at higher magnification in (C) and (D). (E) Dual-immunofluorescence (IF) staining of a thoracic ganglion at 9 dpi for SVV proteins (green) and neural cell adhesion molecule (NCAM; red). Arrows indicate SVV-positive neurons. (F) Dual-IF staining of a thoracic ganglion at 9 dpi for SVV protein (green) and glial fibrillary acidic protein (GFAP; red). Arrow indicates neuron-adjacent SVV-positive cell. (G) Dual-IF staining of a thoracic ganglion from a monkey at 9 dpi for SVV protein (green) and CD3 (red). Arrows indicate SVV-positive T-cells. Asterisks indicate autofluorescent lipofuscin and the borders of the neuronal cell bodies are indicated with dashed lines. (H) Ganglion-derived single-cell suspensions were analyzed by flow cytometry and T-cells were categorized as naive, central memory (CM) and effector memory (EM) T-cells. E–G: nuclei were counterstained with DAPI (blue). ** *p*<0.01 by Mann-Whitney test. B: 200× magnification; C, D: 400× magnification, 2× digital zoom; E: 400× magnification; F, G: 400× magnification, 2× digital zoom.

The hallmark of primary SVV and VZV infection is the capacity of virus to infect and establish latency in ganglionic neurons along the entire neuraxis [1], [13], [39]–[42]. Virus may reach ganglia hematogenously or by retrograde axonal transport along axons innervating varicella lesions [12], [20], [21], [43], [44]. We determined the kinetics of virus infection and the cell types infected in ganglia during primary SVV infection. The SVV DNA load in ganglia was significantly higher in SVV-wt− compared to SVV-EGFP−infected monkeys (*p*<0.01; Mann-Whitney test) (Fig. 7A), peaking at 9 dpi and decreasing thereafter (Fig. 7A), as might be expected during the establishment of latency. Despite high SVV DNA loads, no virus-mediated cytopathology was seen in ganglia (data not shown). Virus antigen was more abundant at 9 dpi than at 13 and 20 dpi (data not shown). SVV-infected cells in ganglia were detected *in situ* by IHC using SVV-specific antiserum (Fig. 7B–D). Dual-IF staining for SVV and the neuron-specific marker NCAM (neural cell adhesion molecule) showed that most SVV^pos^ cells were neurons (Fig. 7E). Occasionally, SVV antigens were seen at the neuronal cell surface or potentially within satellite glial cells (SGC) (Fig. 7D). SGC form a sheet that completely enwraps neuronal cell bodies, providing physical and metabolic support to the neurons and contributing to regulation of the immune response in the peripheral nervous system [45], [46]. Virus-infected cells located in vicinity to neurons did not express the SGC-specific marker glial fibrillary acidic protein (GFAP) [45], implicating that SGC were not infected with SVV at 9, 13 and 20 dpi (Fig. 7F and data not shown). To address the possibility of T-cell–mediated transfer of SVV to neurons, ganglia were examined using dual-IF staining for SVV antigens and CD3. In an SVV-wt−infected monkey euthanized at 9 dpi, SVV-infected T-cells were detected in close proximity to neurons (Fig. 7G). Notably, this animal also had the highest SVV DNA load in blood and ganglia. Flow cytometric analysis of ganglion-derived single-cell suspensions demonstrated that ganglion-resident T-cells were memory T-cells, predominantly EM T-cells (Fig. 7H).

Our findings in ganglia contrast with the pronounced VZV-induced histopathology of both SGCs and neurons found in VZV-infected human fetal ganglia xenografts in the SCID-hu mouse model [12], [47]. Most likely, these differences are due to the use of fetal human ganglia and the lack of adaptive immune responses in the SCID-hu mouse model. The absence of SVV-induced histopathology in ganglia is consistent with previous studies [19], [27] and the inability to recover infectious virus from ganglia [15] at 10 dpi. Nonetheless, virus-induced cytopathology of ganglia may have occurred during the peak of viremia (5–7 dpi), which will be considered in future studies. The detection of SVV protein in the cytoplasm of neurons, but not in the interacting SGC (Fig. 6B–F), supports the notion of retrograde axonal route of virus entry into ganglia [43], [44], [48], [49]. In contrast with this hypothesis, the SVV DNA load did not differ among ganglia, including those that innervated the dermatomes showing varicella rash (Fig. 7A and data not shown). The alternative scenario is that virus traffics to ganglia during viremia within lymphocytes. Indeed, both SVV and VZV enter ganglia before the onset of rash, arguing for hematogenous virus spread [1], [20], [21]. VZV-infected T-cells infiltrate human ganglion xenografts and transmit VZV to neurons in the VZV SCID-hu mouse model [12]. The occasional detection of neuron-interacting, SVV-infected memory T-cells within ganglia (Fig. 7G) supports the role of memory T-cells in virus dissemination to ganglia. Further studies on ganglia from SVV-EGFP−infected monkeys euthanized at earlier times after primary infection are warranted to test this hypothesis.

**Figure 8:**
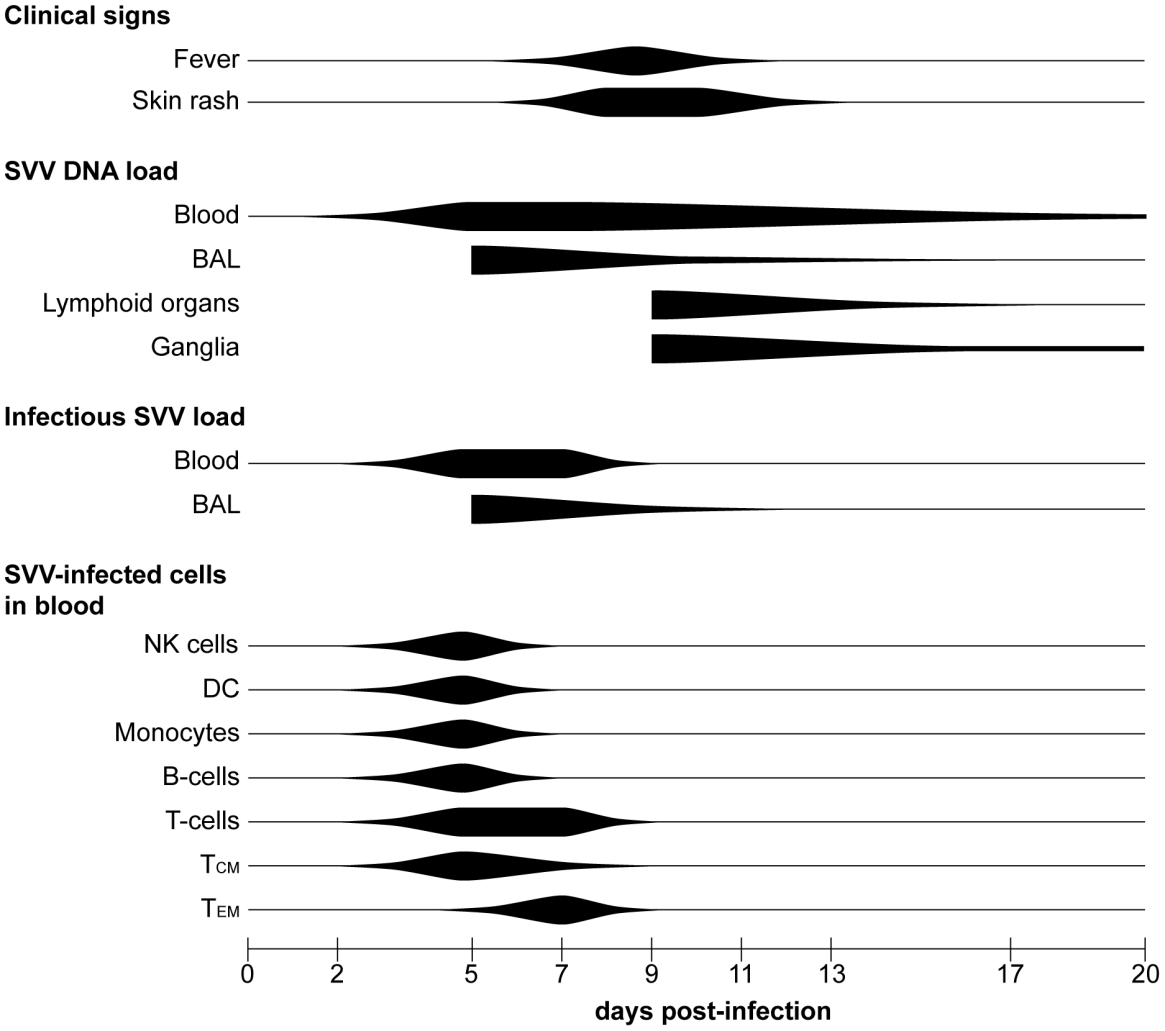
Schematic presentation of primary SVV infection. Figure shows the kinetics of SVV infection and virus-infected cell types in African green monkeys during primary SVV infection. Horizontal lines indicate the time-frame covered by the sampling days. Width of the black bars indicates onset and severity of clinical signs, amount of SVV DNA detected in blood and the sampled organs, and the frequency of SVV-infected cells in peripheral blood during primary SVV infection. Note that BAL samples were obtained no earlier than 5 dpi and animals were euthanized no earlier than 9 dpi. BAL: bronchoalveolar lavage; NK cells: natural killer cells; DC: dendritic cell; T_CM_: central memory T-cells; T_EM_: effector memory T-cells.

**Figure 9:**
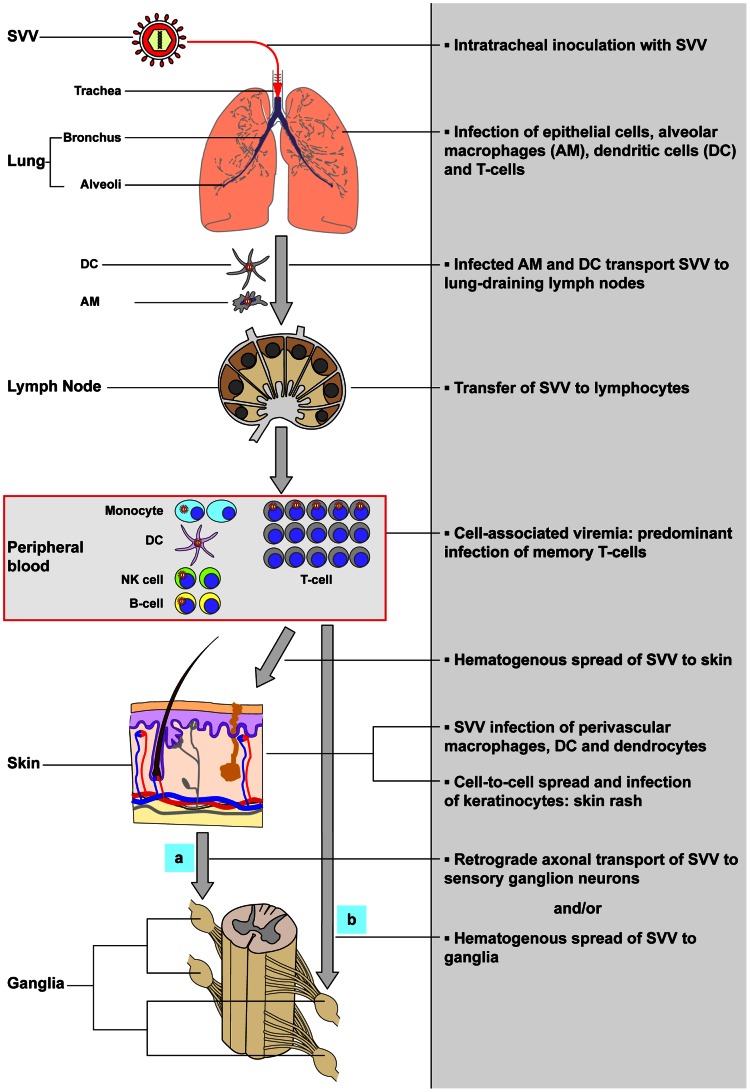
Model of the pathogenesis of primary SVV infection. Upon intratracheal inoculation of African green monkeys, SVV replicates in the lower respiratory tract and infects lung epithelial cells, alveolar macrophages (AM), dendritic cells (DC) and T-cells. SVV-infected AM and DC may transport the virus to draining lymph nodes and subsequently transfer SVV to local lymphocytes resulting in a cell-associated viremia. Memory T-cells are the predominant SVV-infected lymphocyte subset during viremia and may play a central role in dissemination of SVV to its target organs. SVV reaches the skin by the hematogenous route, presumable via virus-infected memory T-cells, which results in the infection of perivascular macrophages, DC and dendrocytes. Subsequently, SVV may infect epidermal and hair follicle keratinocytes via cell-to-cell spread and cause vesicular skin lesions. SVV may enter ganglia by (a) retrograde axonal transport and/or (b) by viremic spread via virus-infected lymphocytes.

The current study is the first to present experimental evidence (summarized in Fig. 8) that supports the role of memory T-cells in the inter-organ dissemination of varicella virus in its natural and immunocompetent host. Our current hypothesis on the pathogenesis of primary SVV infection is presented in Figure 9. We hypothesize that upon intratracheal inoculation, SVV replicates in the respiratory tract and infects epithelial cells, alveolar myeloid cells (AM and/or DC) and T-cells in the lungs. Subsequently, the virus enters the circulation as cell-associated virus predominantly within memory T-cells, first within CM and subsequently within EM T-cells. Most likely, virus-infected alveolar myeloid cells transport SVV to lung-draining lymph nodes, with subsequent transfer of SVV to memory T-cells. Peak viremia coincided with onset of fever and appearance of skin rash. SVV reached the skin by the hematogenous route, most likely via virus-infected memory T-cells. SVV may enter ganglia by retrograde axonal transport from the infected epithelia and/or by the hematogenous route. In addition to memory T-cells, other lymphocyte subsets may also contribute to the viremic spread of SVV. Virus-infected DC, NK cells, B-cells and monocytes were detected in peripheral blood at 5 dpi, albeit at low frequencies compared to memory T-cells. The contribution of each lymphocyte population in transfer of SVV to its target organs will be addressed in future studies by analyzing virus-infected lymphocytes in tissues of animals euthanized during peak viremia at 5–7 dpi.

Like VZV, SVV is considered to spread to naive monkeys via aerosols and therefore most likely targets mucosal epithelial cells of the upper respiratory tract, although – depending on the size of the aerosols – some virus may also directly reach the lower respiratory tract [1], [13], [14], [25]. In the current study, we have used intratracheal inoculation of monkeys with SVV, bypassing the putative initial site of local SVV replication in the upper respiratory tract or tonsils [1], [13], [14]. Primary VZV infections in adults are more severe than in children and frequently complicated by varicella pneumonia [1]. Consequently, the adult status of SVV-infected AGM may have enhanced disease severity, although pneumonia is a common feature in SVV-infected monkeys due to the intratracheal route of inoculation [19], [27]. Recombinant SVV-EGFP was attenuated *in vivo* compared to SVV-wt, possibly due to insertion of the EGFP gene between open reading frames (ORFs) 66 and ORF67 [50]. Recombinant VZV lacking ORF67 is severely impaired for growth in cell culture [51]. Although attenuated in severity, SVV-EGFP–induced disease resembled that of a SVV-wt infection and attenuation did not alter the cell tropism of SVV-EGFP. Both SVV-wt and SVV-EGFP infected the same cell types in lung, lymph nodes, ganglia and skin *in vivo*, and identical PBMC types *in vitro*. The recent cloning of the SVV-wt full-length genome in a bacterial artificial chromosome facilitates the generation of a potentially less attenuated recombinant EGFP-expressing SVV by inserting the EGFP gene adjacent to SVV genes dispensable for growth *in vitro* [51], [52].

Future studies on juvenile African green monkeys, infected with less-attenuated SVV-EGFP strains and via alternative inoculation routes (e.g., via the nose or throat), are warranted. Particularly, analysis of tissues obtained from infected animals euthanized shortly after primary infection are needed to unequivocally determine the early target cell types of SVV, their role in virus dissemination to the target organs affected during primary infection and the route of SVV entry into sensory ganglia [25]. Our current SVV-EGFP/AGM model, which largely covers the clinical and pathological features seen in both SVV-wt−infected monkeys and human varicella patients, provides novel opportunities to elucidate the virus-host cell interactions involved in varicella pathogenesis. This will open new avenues to develop and test new VZV vaccination and therapeutic interventions that limit viremic spread, while inducing long-lasting adaptive VZV-specific immunity.

## Materials and Methods

### Ethics statement

This study was performed in strict accordance with European guidelines (EU Directive on Animal Testing 86/609/EEC) and Dutch legislation (Experiments on Animals Act, 1997). The protocol was approved by the independent animal experimentation ethical review committee DCC in Driebergen, the Netherlands (Erasmus MC permit number EMC2374). Animals were housed in groups, received standard primate feed and fresh fruit daily, and had access to water *ad libitum*. Cages also contained sources of “environmental enrichment” such as hiding places and hanging ropes, tires and other toys. During infection, study animals were housed in HEPA-filtered, negatively pressurized BSL-3 isolator cages. Animal welfare was monitored daily and all animal handling was performed under light anesthesia (ketamine) or deep anesthesia (ketamine and medetomidine) to minimize animal discomfort. After deep anesthesia, atipamezole was administered to antagonize the effect of medetomidine. Animals were euthanized by sedation with ketamine and medetomidine followed by exsanguination.

### Viruses

Low-passage clinical isolates of the Delta herpesvirus strain of SVV-wt and SVV-EGFP were obtained from PBMC of acutely infected AGM and propagated less than 5 times in AGM- kidney epithelial cell line BSC-1 (American Tissue Type Culture no. CCL-26) to generate virus stocks as described [53]. Virus stocks were confirmed as *Mycoplasma*-free. SVV-EGFP was generated by insertion of the EGFP gene downstream from a Rous sarcoma virus promoter between SVV ORF66 and ORF67 [20], [50].

### SVV infection of PBMC *in vitro*

PBMC from SVV-naive AGM were infected by co-cultivating PBMC (5×10^5^) with SVV-EGFP–infected Vero cells (0.5–1×10^5^), showing 70% virus-induced cytopathic effect (CPE), in 0.5 ml DMEM supplemented with antibiotics and 10% heat-inactivated fetal bovine serum (FBS) for 24 hr in 24-well plates at 37°C in a CO_2_-incubator. Mock-infected PBMC were similarly generated by co-cultivating PBMC with uninfected Vero cells. SVV-EGFP−infected PBMC were stained and analyzed by flow cytometry or spotted on microscope slides, fixed and stained by immunofluorescence for SVV as described below.

### Experimental SVV infection of AGM, necropsy and collection of tissues

Five adult (10- to 12-year-old) SVV-seronegative AGMs (*Cercopithecus aethiops*) with intraperitoneal implanted temperature transponders were inoculated intratracheally with ∼10^6^ plaque-forming units (pfu) of SVV-EGFP (n = 3 animals; 1 male and 2 females) or SVV-wt (n = 2 animals; 1 male and 1 female) diluted in 5 ml of phosphate-buffered saline [21]. Just before infection, animals were sedated with ketamine and medetomidine. The abdomen and back of the animals were shaved to allow careful examination for skin rash every other day until necropsy. Heparinized blood samples were collected under light ketamine sedation at 0, 2, 7, 11, 13, 17 and 20 dpi. Bronchoalveolar lavage (BAL) samples and peripheral blood (PB) samples were collected under deep anesthesia at 5 and 9 dpi. Three punch biopsies (3 mm) of varicella rashes and EGFP fluorescent skin tissue, while showing no characteristic varicella-like skin rash by the naked eye, were obtained from anesthetized animals at 9 dpi under anesthesia. SVV-EGFP−infected animals were checked for macroscopic EGFP fluorescence using a custom-made lamp containing 6 LEDs (peak emission 490–495 nm) mounted with D480/40 bandpass filters [22]. Fluorescence was detected by an amber cover of a UV transilluminator used for screening DNA gels [22]. Photographs were taken using a Nikon D80 SLR camera. SVV-infected animals were euthanized at 9 dpi (n = 2; one SVV-wt- and one SVV-EGFP−infected animal), 13 dpi (n = 2; one SVV-wt− and one SVV-EGFP−infected animal) and 20 dpi (one SVV-EGFP-infected animal). Multiple tissues including lung, lymph nodes, spleen, tonsils, skin and ganglia were collected at necropsy and either snap-frozen or fixed and paraffin-embedded.

### Collection and processing of PB and BAL samples

PBMC were isolated by density-gradient centrifugation and used for virus isolation, DNA isolation and flow cytometry or cryopreserved as viable cells as described [19]. Cells recovered from BAL samples were centrifuged, dissolved in RPMI-1640 medium supplemented with 10% FBS plus antibiotics (R10F medium), and used for virus isolation, DNA isolation and flow cytometry as described [22].

### Virus isolation from PB and BAL samples

Infectious SVV was isolated from PB and BAL cells by incubating 1–2×10^6^ cells in 10-fold serial dilutions in R10F medium on confluent monolayers of BSC-1 cells in 6-well plates. Cells were monitored for SVV-induced CPE or EGFP expression after 7 days of co-cultivation and results were expressed as numbers of SVV-infected cells per 10^6^ input PBMC and BAL cells.

### Nucleic acid extraction and quantitative PCR (qPCR)

DNA was isolated from PBMC, BAL cells, pooled ganglia, pooled lymph nodes, tonsils and spleen using a QIAamp DNA Mini Kit (Qiagen). qPCR was performed in triplicate on a ABI Prism 7500 using Taqman 2× PCR Universal Master Mix (Applied Biosystems) with primers and probes specific for SVV open reading frame 21 (ORF21) and the pan-primate single-copy gene oncostatin-M (OSM) as described [14], [21], [54]. DNA dilutions obtained from uninfected PBMC were used to validate the OSM Taqman assay.

### Flow cytometry

PBMC were either directly used for flow cytometry to detect EGFP+ cells or stained for indicated markers using fluorochrome-conjugated mAbs: CD3^APC-Cy7^ (clone SP34-2), CD4^AmCyan^ (L200), CD8^PerCp^ (SK1), CD14^PE^ (M5E2), CD16^AF647^ (3G8), CD20^PE-Cy7^ (L27) and HLA-DR^PacificBlue^ (L243) (all from BD Biosciences) to delineate SVV-infected PBMC subsets. To identify SVV-infected T-cell subtypes, PBMC from infected AGMs were stained with mAbs specific for CD3^APC-Cy7^ (SP34-2), CD4^PacificBlue^ (L200), CD8^MCyan^ (SK1), CD28^APC^ (28.2), CD95^PerCp^ (DX2), CCR4^PE-Cy7^ (1G1) (all from BD Biosciences) and CD137^PE^ (4B4-1; Miltenyi biotec). T-cells were categorized into naive, central memory (CM) and effector memory (EM) T-cells based on differential expression of CD28 and CD95 (Fig. S2) [28]. In contrast to humans and macaque species, AGMs have three distinct CD3^pos^ T-cell populations based on expression of CD4 and CD8α: CD4^pos^CD8α^neg^ (CD4^pos^), CD4^neg^ CD8α^dim^ (CD8^dim^) and CD4^neg^CD8α^bright^ (CD8^bright^) (Fig. S2)[28]. BAL cells were stained as described for PBMC, except for inclusion of anti-CD45^APC^ (MB4-6D6; Miltenyi biotec) instead of anti-CD16 mAb. Fluorescence was detected on a FACS Canto II and analyzed using FACS Diva software (BD Biosciences). At least 10^6^ viable cells were measured to accurately identify EGFP^pos^ cells.

### *In situ* analyses

Immunohistochemical and immunofluorescence staining was performed using predefined optimal dilutions of primary mAbs directed against: CD3 (clone F7.2.38; Dako), CD11c (NCL-L-CD11c-563; Novocastra), CD20 (L26; Dako), CD68 (KP1; Dako), NCAM (123C3.D5; Thermo Fischer Scientific), GFAP (4A11; BD Biosciences), keratin (AE1/AE3; Thermo Fischer Scientific), α-smooth muscle actin (1A4; Sigma-Aldrich) and rabbit polyclonal antibodies directed against GFP (IgG fraction; Invitrogen) and SVV nucleocapsid proteins [19]. As isotype controls, sections were incubated with mouse IgG1, IgG2a and IgG2b and rabbit immunoglobulins (Dako). Paraformaldehyde-fixed (4%), paraffin-embedded tissue sections were deparaffinized, rehydrated, subjected to heat-induced antigen retrieval in citrate buffer (10 mM, pH = 6.0), blocked and incubated with primary antibodies overnight at 4°C as described [46], [55]. Immunohistochemical staining was visualized using the avidin-biotin system (Dako) in combination with 3-amino-9-ethylcarbazole (AEC) (Sigma-Aldrich) and sections were counterstained with hematoxylin (Sigma-Aldrich) as described [46], [55].

For immunofluorescence staining, sections were incubated with secondary Alexa Fluor 488 (AF488)- or AF594-conjugated goat-anti-mouse and/or goat-anti-rabbit antibodies and mounted in Prolong Gold Antifade reagent with 4′,6-diamidino-2-phenylindole (Invitrogen) [56]. Sections were analyzed on a Zeiss LSM 700 confocal laser scanning microscope fitted on an Axio Observer Z1 inverted microscope (Zeiss). Images were obtained using 2–4× frame averaging and the pinhole adjusted to 1 airy unit. ZEN 2010 software (Zeiss) was used to adjust brightness and contrast.

## Supporting Information

Figure S1 **Gating strategy for flow cytometric differentiation of bronchoalveolar lavage (BAL) cells of African green monkeys.** BAL cells were gated on viable cells based on forward scatter (FSC) and sideward scatter (SSC) properties and defined as CD45^neg^ cells or CD45^pos^ leukocytes. CD45^pos^ BAL leukocyte subsets were defined as follows: CD3^neg^CD20^neg^MHC-II^pos^CD14^pos/dim^ = alveolar macrophages (AM) or dendritic cells (DC); CD20^pos^MHC-II^pos^ = B-cells; CD3^pos^T-cells; CD4^neg^CD8α^high^ = CD8^bright^ T-cells, CD4^neg^CD8α^dim^ = CD8^dim^ T-cells, and CD4^pos^CD8α^neg^ = CD4^pos^ T-cells.
(TIF)

Figure S2 **Gating strategy for flow cytometric differentiation of PBMC subsets from African green monkeys.** (A) Viable lymphocytes were selected based on forward scatter (FSC) and sideward scatter (SSC) properties and PBMC subsets were defined as follows: CD3^pos^CD16^neg^ = T-cells; CD3^neg^CD16^pos^ = natural killer (NK) cells; CD3^neg^CD14^pos^MHC-II^pos^ = monocytes; CD20^pos^MHC-II^pos^ = B-cells; CD3^neg^CD20^neg^CD14^neg^CD16^neg^MHC-II^pos^ = dendritic cells (DC). (B) AGM-specific T-cell subsets were categorized based on the expression of CD8α and CD4: CD4^neg^CD8α^high^ = CD8^bright^ T-cells, CD4^neg^CD8α^dim^ = CD8^dim^ T-cells, and CD4^pos^CD8α^neg^ = CD4^pos^ T-cells. (C) Based on the differential expression of CD28 and CD95, T-cells were categorized as naive (CD28^pos^CD95^neg^), central memory (CM; CD28^pos^CD95^pos^) and effector memory (EM; CD28^neg^CD95^pos^) T-cells.
(TIF)

Figure S3 **Peripheral blood CCR4^pos^ and CD137^pos^ T-cells were not preferentially infected in African green monkeys.** Flow cytometric detection of EGFP expression in central memory (CM) and effector memory (EM) T-cells at 5 dpi (A) and 7 dpi (B). Gating strategy was according to Figure S2. Data are given as means ± SEM.
(TIF)

Figure S4 **Memory T-cells were preferentially infected** ***in vitro*** **.** (A) SVV-naive African green monkey peripheral blood mononuclear cells (PBMC) were infected with SVV-EGFP *in vitro* and stained 24 hr later for SVV proteins to show that EGFP fluorescence (green) co-localized with SVV proteins (red). Nuclei were counterstained with DAPI (blue). Magnification: 400×. (B) African green monkey PBMC were infected with SVV-EGFP *in vitro* and analyzed 24 hr later by flow cytometry for EGFP expression in the indicated lymphocyte subsets. Data are plotted as the frequency of EGFP^pos^ cells within individual PBMC subsets (within subset) or as the percentage of EGFP^pos^ cells within each lymphocyte subset relative to the total number of PBMC (absolute). (C, D) Percentage of EGFP^pos^ cells in the indicated T-cell subsets as assessed by flow cytometry. The lymphocyte subsets were defined as described in Figure S2. Data represent means ± SEM of three independent experiments performed on PBMC from three animals. * *p*<0.05 by Mann-Whitney test.
(TIF)
