# Supplementary figures and images for: T-Cell Tropism of Simian Varicella Virus during Primary Infection
Source: PLoS Pathog. 2013 May 9;9(5):e1003368. doi: 10.1371/journal.ppat.1003368 (PMC3649965; doi:10.1371/journal.ppat.1003368)

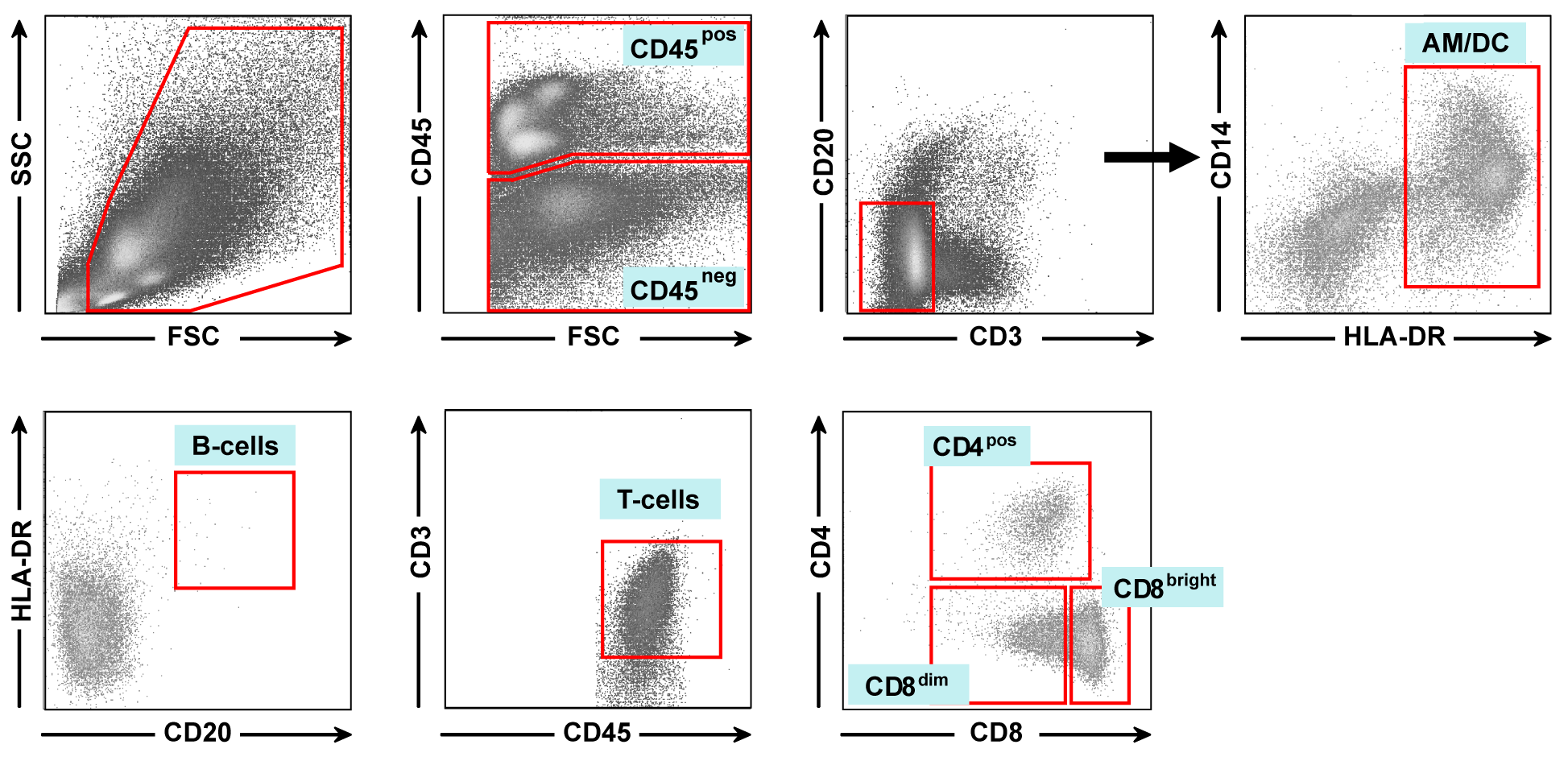

Supplement: Figure S1 — Gating strategy for flow cytometric differentiation of bronchoalveolar lavage (BAL) cells of African green monkeys. BAL cells were gated on viable cells based on forward scatter (FSC) and sideward scatter (SSC) properties and defined as CD45neg cells or CD45pos leukocytes. CD45pos BAL leukocyte subsets were defined as follows: CD3negCD20negMHC-IIposCD14pos/dim = alveolar macrophages (AM) or dendritic cells (DC); CD20posMHC-IIpos = B-cells; CD3posT-cells; CD4negCD8αhigh = CD8bright T-cells, CD4negCD8αdim = CD8dim T-cells, and CD4posCD8αneg = CD4pos T-cells. (TIF) [file ppat.1003368.s001.tif]

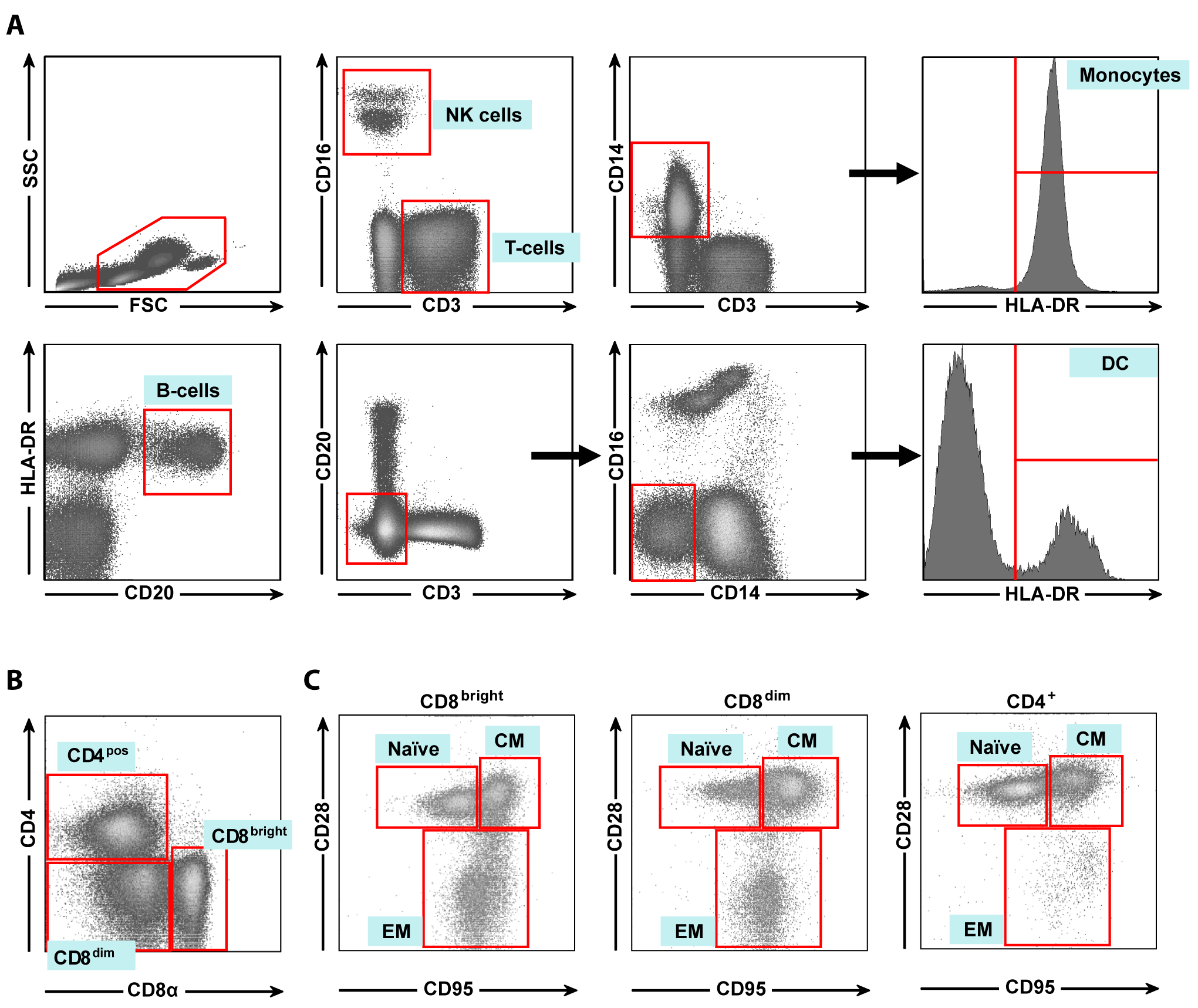

Supplement: Figure S2 — Gating strategy for flow cytometric differentiation of PBMC subsets from African green monkeys. (A) Viable lymphocytes were selected based on forward scatter (FSC) and sideward scatter (SSC) properties and PBMC subsets were defined as follows: CD3posCD16neg = T-cells; CD3negCD16pos = natural killer (NK) cells; CD3negCD14posMHC-IIpos = monocytes; CD20posMHC-IIpos = B-cells; CD3negCD20negCD14negCD16negMHC-IIpos = dendritic cells (DC). (B) AGM-specific T-cell subsets were categorized based on the expression of CD8α and CD4: CD4negCD8αhigh = CD8bright T-cells, CD4negCD8αdim = CD8dim T-cells, and CD4posCD8αneg = CD4pos T-cells. (C) Based on the differential expression of CD28 and CD95, T-cells were categorized as naive (CD28posCD95neg), central memory (CM; CD28posCD95pos) and effector memory (EM; CD28negCD95pos) T-cells. (TIF) [file ppat.1003368.s002.tif]

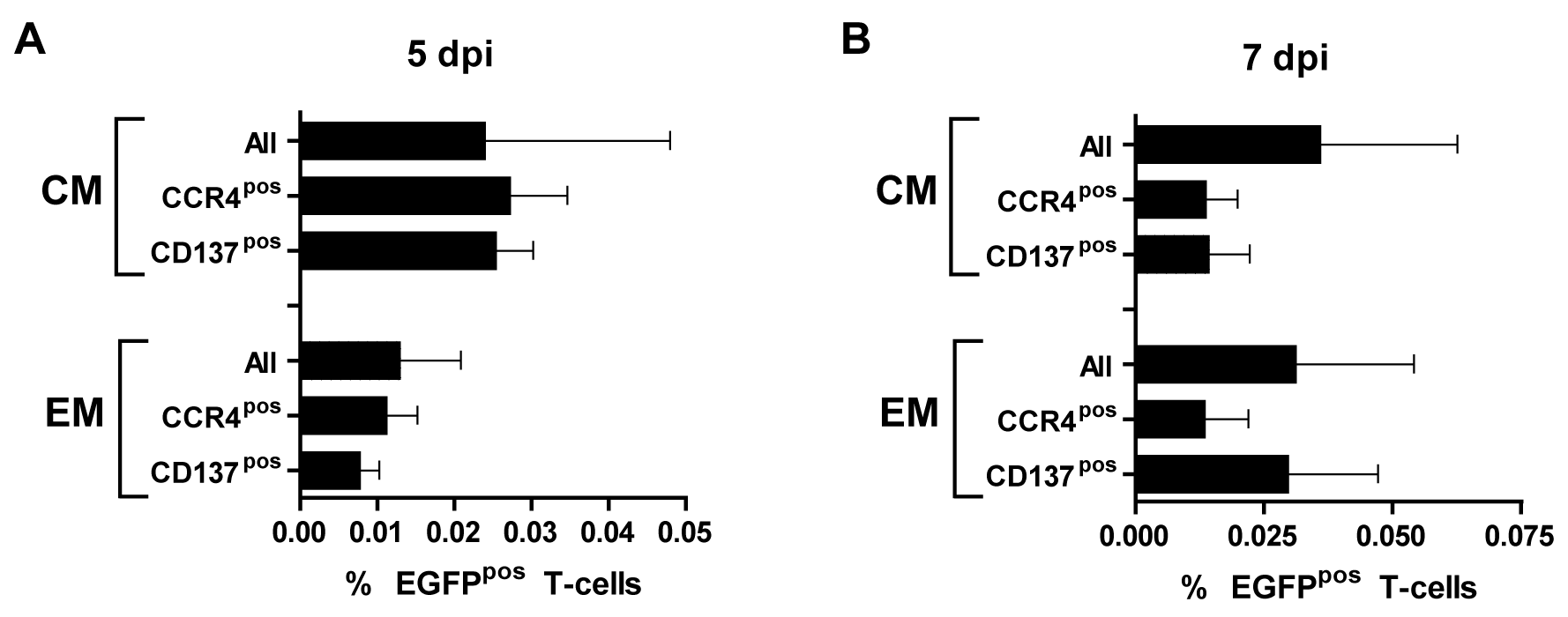

Supplement: Figure S3 — Peripheral blood CCR4pos and CD137pos T-cells were not preferentially infected in African green monkeys. Flow cytometric detection of EGFP expression in central memory (CM) and effector memory (EM) T-cells at 5 dpi (A) and 7 dpi (B). Gating strategy was according to Figure S2. Data are given as means ± SEM. (TIF) [file ppat.1003368.s003.tif]

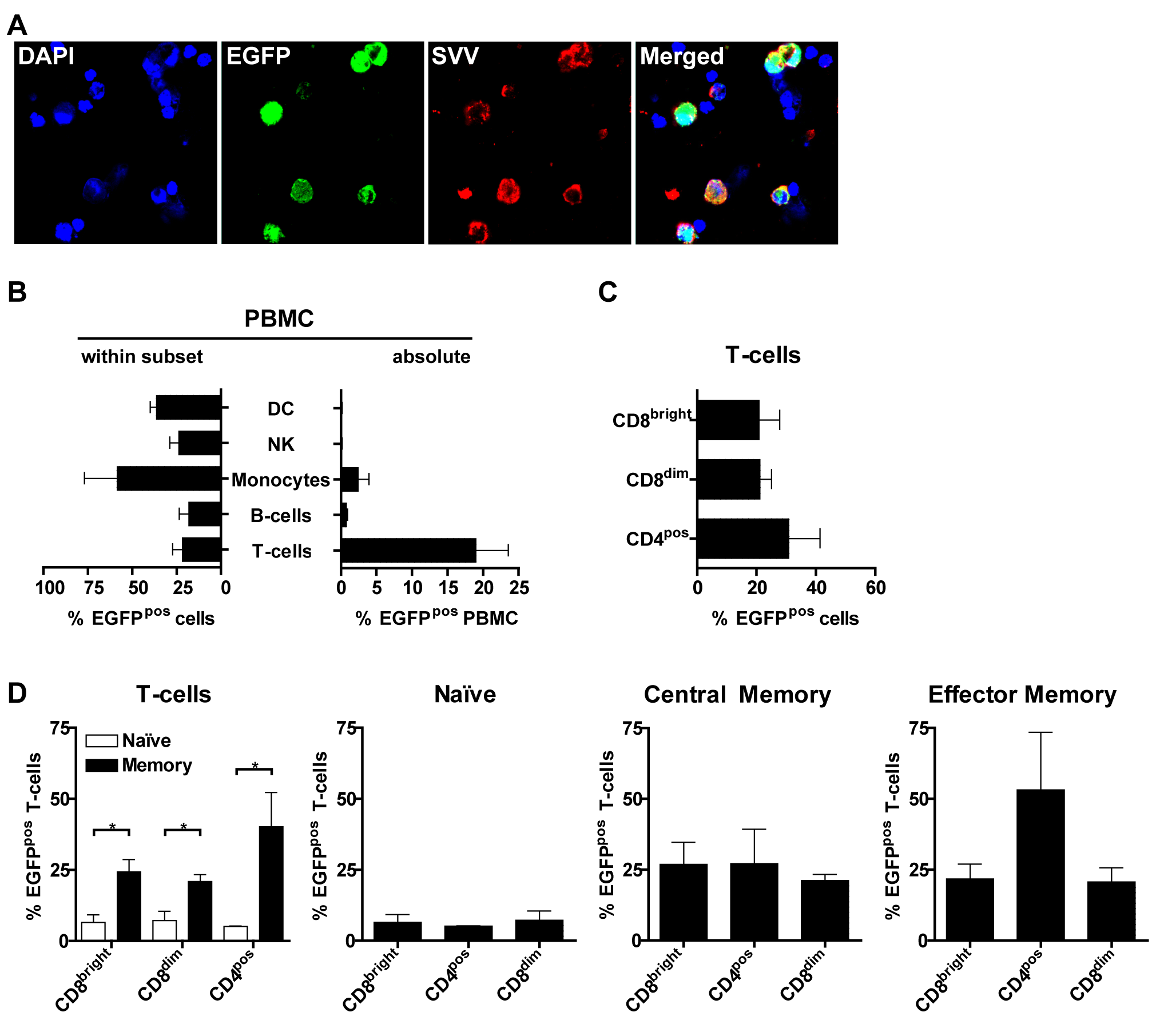

Supplement: Figure S4 — Memory T-cells were preferentially infected in vitro . (A) SVV-naive African green monkey peripheral blood mononuclear cells (PBMC) were infected with SVV-EGFP in vitro and stained 24 hr later for SVV proteins to show that EGFP fluorescence (green) co-localized with SVV proteins (red). Nuclei were counterstained with DAPI (blue). Magnification: 400×. (B) African green monkey PBMC were infected with SVV-EGFP in vitro and analyzed 24 hr later by flow cytometry for EGFP expression in the indicated lymphocyte subsets. Data are plotted as the frequency of EGFPpos cells within individual PBMC subsets (within subset) or as the percentage of EGFPpos cells within each lymphocyte subset relative to the total number of PBMC (absolute). (C, D) Percentage of EGFPpos cells in the indicated T-cell subsets as assessed by flow cytometry. The lymphocyte subsets were defined as described in Figure S2. Data represent means ± SEM of three independent experiments performed on PBMC from three animals. * p<0.05 by Mann-Whitney test. (TIF) [file ppat.1003368.s004.tif]
